# Acceptor formation in Mg-doped, indium-rich Ga_*x*_In_1−*x*_N: evidence for p-type conductivity

**DOI:** 10.1186/1556-276X-7-574

**Published:** 2012-10-18

**Authors:** Naci Balkan, Engin Tiras, Ayse Erol, Mustafa Gunes, Sukru Ardali, MCetin Arikan, Dalphine Lagarde, Helene Carrère, Xavier Marie, Cebrail Gumus

**Affiliations:** 1 Department of Computer Science and Electronic Engineering, University of Essex, Colchester, CO4 3SQ, UK; 2 Department of Physics, Anadolu University, Eskişehir, 26470, Turkey; 3 Faculty of Science, Department of Physics, Istanbul University, Vezneciler, Istanbul, 34134, Turkey; 4 Université de Toulouse, LPCNO, INSA-UPS-CNRS, 135 Avenue de Rangueil, Toulouse, 31077, France; 5 Department of Physics, Cukurova University, Adana, 01330, Turkey

## Abstract

We report on the Mg-doped, indium-rich Ga_*x*_In_1−*x*_N (*x* < 30). In the undoped material, the intrinsic electron density is very high and as a result there is no detectable photoconductivity (PC) signal within the range of temperatures of 30 <*T* < 300 K. In the Mg-doped material however, where the conductivity is reduced, there is a strong PC spectrum with two prominent low-energy peaks at 0.65 and 1.0 eV and one broad high-energy peak at around 1.35 eV. The temperature dependence of the spectral photoconductivity under constant illumination intensity, at *T* > 150 K, is determined by the longitudinal-optical phonon scattering together with the thermal regeneration of non-equilibrium minority carriers from traps with an average depth of 103 ± 15 meV. This value is close to the Mg binding energy in GaInN. The complementary measurements of transient photoluminescence at liquid He temperatures give the e-A^0^ binding energy of approximately 100 meV. Furthermore, Hall measurements in the Mg-doped material also indicate an activated behaviour with an acceptor binding energy of 108 ± 20 meV.

## Background

During the last decade, there has been intense research activity in indium-rich GaInN. The reason for the interest lies, primarily, in the unusual optoelectronic properties of these semiconductors, where alloying InN with AlN and GaN extends the operational spectrum of the nitride-based optoelectronic devices from the ultraviolet to the near infrared region covering wavelengths from 200 to 1,650 nm [1-3]. Until a few years ago, the commonly accepted value for the InN band gap energy was 1.89 eV [4], but the majority of the results were obtained on samples grown by sputtering technique and were characterised as having a polycrystalline structure. Moreover, most of the theoretical work reported at the time was based on this wrong value for the band gap. In 2001 Inushima et al. reported a much lower value of *E*_G_ = 1.1 eV for the molecular beam epitaxy (MBE)-grown samples [5]. Davydov et al. [6] performed optical absorption and photoluminescence measurements and reported band gap energy of 0.9 eV. Further improvement in the growth techniques has led to the availability of high quality material. Wu et al. [7] found a value for the band gap between 0.7 and 0.8 eV from their photo-modulated reflectance and photoluminescence (PL) measurements. Since then a large number of papers from different research groups have been published reporting a value between 0.65 and 0.9 eV in the MBE-grown material. It is interesting to note that none of these groups could detect any peak at 1.9 eV. The band gap variation with temperature has been addressed in some publications with no apparent overall agreement on the actual behaviour. Wu et al. [7] showed a slight increase in energy with increasing temperature, which is in contrast with the behaviour of most direct semiconductors. However, Davydov et al. [8] reported a more orthodox dependence, with a reduction of 23 meV in energy when the temperature is increased from 77 to 300 K.

The band gap energy is sensitive to carrier concentration, a characteristic which is typical in a degenerate semiconductor (Burstein-Moss shift). Davydov et al. found an empirical relation between energy gap *Eg* and carrier concentration as given by [9]

(1) $$E\text{g}=0.65+0.0166\;\left(\;m_{0}/m^{*}\right)\;\left(\;n\times 10^{-19}\right)\;{\;}^{2/3}\text{.}$$

Here, *m*_0_, m*, and *n* are the electron mass, GaInN effective mass and carrier concentration respectively. Since the revision of its band gap, the work on InN and indium-rich GaInN has been primarily focused on their optical properties, and only very limited work on their electrical properties has been reported [2,3].

As-grown InN epilayers show n-type behaviour which is independent of the growth technique. The situation is similar to the case of GaN, and analogous explanations have been tentatively given to account for the background carrier concentration [1-10]. The reduction in the background carrier density and increase in the mobility were achieved recently, and electron densities as low as 4 × 10^19^ cm^−3^ were obtained with metalorganic vapour phase epitaxy (MOVPE) [11]. However, the best results in terms of electron density and carrier mobility were achieved using MBE. It has also been shown that the Hall mobility increases with increasing layer thickness in both MBE and MOVPE samples. This is believed to be due to the reduction in the defect density with increasing thickness [12]. Higashiwaki and Matsui [13] found, however, that the improvement in Hall mobility with increasing thickness is valid up to a critical thickness value of 150 nm, above which the mobility is approximately constant. It was recently shown that irradiation of InN films with 2 MeV He+ ions followed by thermal annealing below 500°C creates films with high electron concentrations and mobilities [14]. Recently, Lu et al. [15] have reported that the Hall carrier density does not approach zero as the layer thickness decreases. In order to explain their observations, they suggested the existence of more than one conducting layer, one of which was a surface charge layer, to explain the capacitance-voltage measurements [15]. This hypothesis was also confirmed by high-resolution electron energy loss spectroscopy [16-18]. The work shows clearly the surface electron accumulation which is interpreted as the consequence of donor-type surface states pinning the surface Fermi level above the conduction band minimum.

There have been few early attempts to dope indium-rich GaInN and InN p-type using Mg, but they were unsuccessful [19]. The high electron concentration requires a high Mg doping level to achieve compensation. However, increasing Mg concentration results in a higher defect density. This degrades the surface morphology and crystal quality. Furthermore, the presence of an n-type surface conducting channel screens out the effect of doping of the bulk layer with Mg. However, Jones et al. have recently demonstrated p-type doping using capacitance-voltage (*C-V*) profiling measurements [20]. They also observed a small photoconductivity signal in Mg-doped GaInN in the entire composition range [21]. Further evidence for successful p doping of In-rich GaInN has been reported by various groups recently [22,23].

The current work presented here is, to our knowledge, the first study of spectral photoconductivity in Mg-doped, indium-rich GaInN and InN. The photoconductivity (PC) results, coupled with the transient PL and Hall measurements, provide direct evidence for the formation of p-type conducting channels in Mg-doped GaInN.

## Methods

We have studied the Hall effect in undoped and Mg doped samples in darkness and investigated the spectral photoconductivity and photoluminescence in indium-rich GaInN grown by MBE. The temperature dependence of the DC conductivity in Mg-doped GaInN is explained using a multi-channel conduction mechanism, involving p-doped layers in parallel with the n layer. Temperature dependence of photoconductivity is analysed using the trap dynamics of photo-generated carriers in conjunction with optical phonon-assisted momentum relaxation mechanism. Complementary measurements of the transient photoluminescence at low temperatures are carried out to study e-A transitions in Mg-doped GaInN.

## Results and discussion

The samples were grown by a Varian GEN-II gas-source MBE system on (0001) sapphire substrates with a few hundred nanometre-thick AlN buffer layer. This was followed by the growth of Ga_*x*_In_1−*x*_N epilayers at around 470°C with a growth rate of approximately 0*.*6 μm*/*h. The undoped sample (GS 1509) had 30% Ga and the Mg-doped one (GS1989) had 20% Ga. The other undoped sample which was used in the PL measurements (GS 1508) had a similar composition to the Mg-doped sample.

### Hall measurements

Hall measurements were carried out in darkness in the temperature range between *T* = 9 and 300 K. The current flowing through the sample was kept relatively low (*I* < 50 μA) to ensure ohmic conditions. Both the mobility and carrier concentration were found to be independent of current and magnetic field at all temperatures.

**Figure 1:**
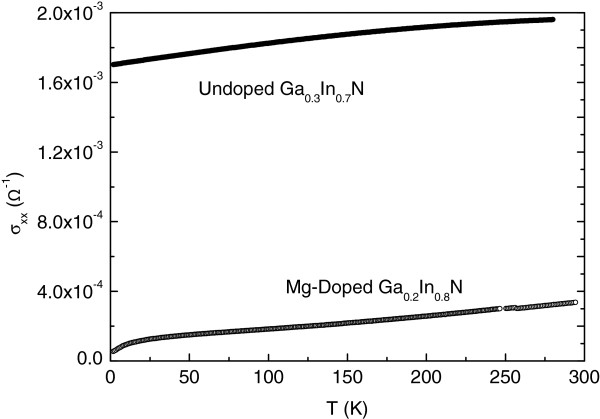
**The temperature dependence of the conductivity for the undoped (GS 1509) and Mg**-**doped (GS1989) samples.**

The temperature dependence of the dark conductivity (DC) for the undoped (GS 1509) and Mg-doped (GS 1989) samples are shown in Figure 1. It is clear that the room temperature conductivity in the Mg-doped sample is about an order of magnitude smaller than that in the undoped material (at 2 K it is two orders of magnitude smaller).

**Figure 2:**
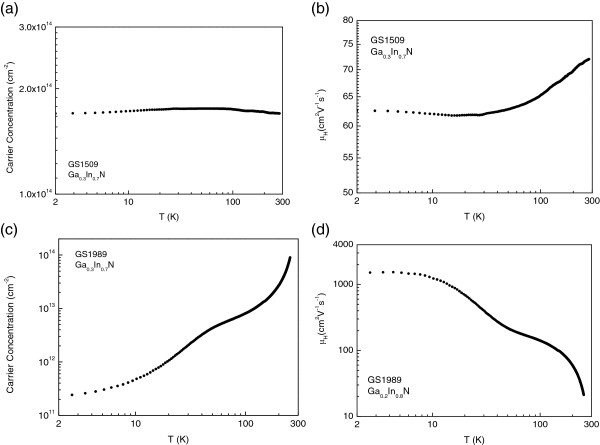
Hall carrier density and Hall mobilities for the undoped (a, b) and Mg-doped materials (c, d).

The weak temperature dependence of both Hall density and mobility in the undoped material GS1509 that is shown in Figure 2a,b is a typical feature in undoped GaInN (and InN). The Hall carrier density at *T* = 2 K is *n* = 1.7 10^14^ cm^−2^ and it changes only by about 5% in the whole temperature range between 2 and 300 K. The parallel conductivity from the degenerate surface layer results in very small temperature sensitivity [1-3]. The electron mobility, as plotted in Figure 2b which is *μ* = 63 cm^2^/Vs at *T* = 2 K and *μ* = 72 cm^2^/Vs at *T* = 300 K, i.e., has a very weak temperature dependence. This temperature dependence has also been observed by other research groups and explained as either being due to a reduced electron–phonon interaction or high degeneracy [24]. Since InN which is similar to the other group III-N semiconductors is a highly polar material, scattering due to polar optical phonons is expected to play an important role. Therefore, the observed weak temperature dependence is more likely to be associated with the latter, i.e. the screening of polar interaction with high carrier density [25,26].

The temperature behaviour of Hall data which is similar to ours is commonly interpreted in the literature in terms of two parallel conducting channels as described by Jones et al. [20] and Mahboob et al. [16,17] as an electron accumulation layer on the surface and a 3-D bulk layer. However, variable magnetic field Hall measurements and the quantitative mobility spectrum analysis [27] of the Hall data must be considered in order to distinguish the contribution of each channel though the analysis which may be further complicated as a result of the small discrepancy between the electron densities and the mobilities in the two channels [28].

**Figure 3:**
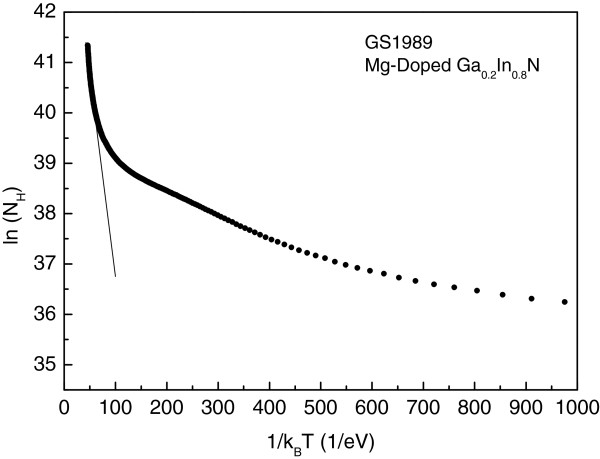
**Logarithm of the Hall carrier density versus inverse temperature.** The carrier density is thermally activated with an activation energy of *E*_a_ = 108 ± 20 meV (from the slope of the line going through the experimental data at highest measurement temperatures).

**Figure 4:**
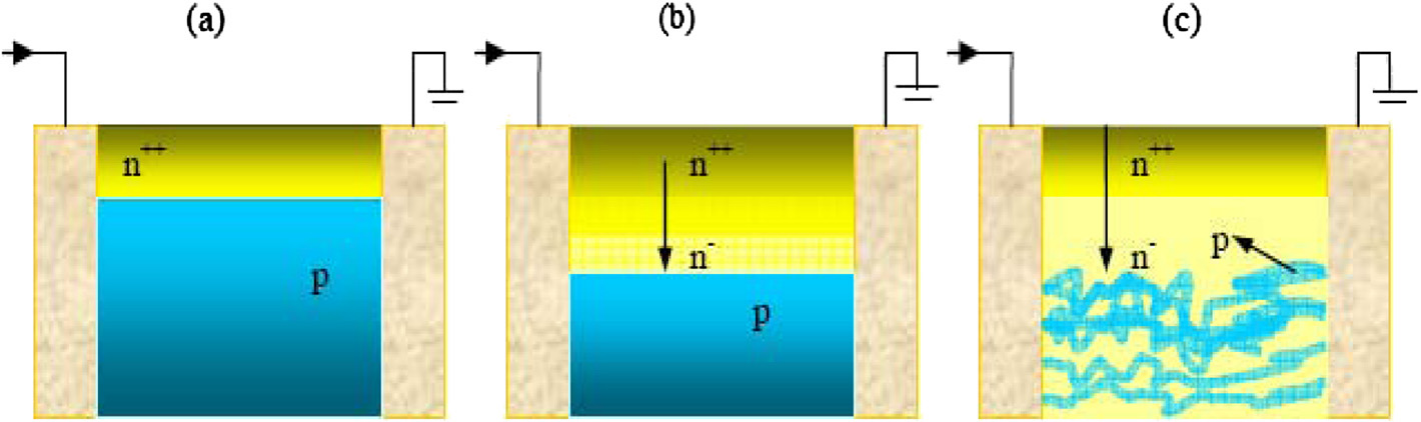
**Schematic of the possible conduction channels in Mg-doped GaInN.** (in all the mechanisms the surface layer is n-type degenerate) (**a**) Bulk layer is p-type. (**b**) The p-type conductivity occurs in the bulk layer below a certain depth from the surface [20,29]. (**c**) The p-type conductivity is not within a uniform layer but occurs through p-type percolation channels [30,31].

The temperature dependence of the Hall density and mobility for the Mg-doped material (GS1989) is shown in Figure 2c,d. Electron mobility decreases with increasing temperature from 0.16 m^2^V^−1^ s^−1^ at *T* = 2 K to 0.003 m^2^V^−1^ s^−1^ at *T* = 300 K, while the carrier density changes considerably more than that in the undoped material from around 2 × 10^11^ to 9.0 × 10^13^ m^−2^ in the same temperature range. We have plotted the logarithm of the Hall carrier density as a function of inverse temperature in Figure 3. There is a clear tendency to an activated behaviour at high temperatures. This observation may be explained speculatively, in terms of a mechanism involving two-channel conductivity with both types of type carriers, namely: (1) the 2DEG accumulation at the surface layer where the density for these carriers will be temperature independent as it is the case in undoped material as shown in Figure 2a and (2) the bulk layer. For the bulk conductivity, we may assume either of the equally plausible three scenarios: (1) The whole bulk layer is p-type or (2) p-type conductivity occurs in the bulk layer only below a certain depth from the surface and adjacent to the bottom buffer layer as suggested by Jones et al. and Ager et al. [20,29]. Alternatively, (3) p-type conductivity is not within a uniform layer but occurs through p-type percolation channels around slightly compensated n-type islands, the sizes of which may vary, depending on the local electron and hole densities, from nano to microscale islands or chain (similar to the model that was proposed by us and Ridley [30]) to explain successfully hot electron percolation in 2-D GaAs and superconductivity in InN [31]. These three mechanisms are depicted schematically in Figure 4. In either of the three models, at high temperatures as the Mg acceptors [1,32] are ionized, the contribution of thermally activated holes to the conductivity increases as observed. From Figure 3 at the highest measurement temperatures, we obtain an activation energy close to Δ*E* = 108 ± 20 meV. This value is within the range of the reported binding energy of Mg in indium-rich GaInN and InN [22]. It should be noted that the accuracy of the activation energy obtained from the temperature dependence of the Hall density in our limited range of high temperatures is within about ±20%.

Following the analysis for parallel conduction by Kane et al. [33] and considering the above model for the bulk and surface conductivity channels in Mg-doped GaInN [1,32], the expressions for the effective Hall mobility and carrier density due to the two n type and p-type conducting channels may be written as

(2) $$n_{H}=\frac{{\left(n_{e}\mu_{e}+p\mu_{p}\right)}^{2}}{n_{e}\mu_{e}^{2}+p\mu_{p}^{2}}$$

(3) $$\mu_{H}=\frac{n_{e}\mu_{e}^{2}+p\mu_{p}^{2}}{n_{e}\mu_{e}+p\mu_{p}}\text{,}$$

where *n*_e_ and *μ*_e_*,* are the electron density and the mobility in the GaInN accumulation layer, and *p* and *μ*_p_ are the hole density and mobility in the p-channels of the bulk layer. Here it is assumed that the electron density in the n-type regions in the bulk (*n*_B_) is negligible compared to the surface accumulation density (*n*_B_*<< n*_e_).

The temperature dependences of *n*_e_ and *μ*_e_ in the undoped material are expected to be weak as it is observed by us in Figure 2a,b and by others [24]. Therefore, we can assume that the temperature dependence of the Hall carrier density and mobility in the Mg-doped sample is fundamentally due to the temperature dependence of both *p,* and *μ*_p_ in the p-channels in the bulk layer. Furthermore, at low temperatures, because of the freeze-out of the free hole density, *n*_e_*> > p* and the conductivity are fundamentally due to the conduction in the accumulation layer, i.e.

(4) T→0

(5) $$\mu H=\mu_{e\;}and\;n_{H}=n_{e}\text{.}$$

At higher temperatures according to Equations 1 and 2, the measured Hall carrier density and mobility will be the complicated functions of temperature as determined by the temperature dependence of the effective scattering mechanisms in the accumulation layer and the bulk. However, the temperature dependence of the Hall density will be primarily due to the thermally activated behaviour of the holes as we have observed in Figure 3. As a result the activation energy obtained from Figure 3 should correspond to the Mg binding energy.

### Spectral photoconductivity

Since the conductivity in the p-type material is about a factor of 100 smaller than the corresponding undoped material, it is possible to have a detectable spectral PC in the Mg-doped material. We carried out the PC spectrum measurements on both the undoped and the Mg-doped samples at temperatures between *T* = 33 and 300 K. In the measurements a focused beam of light from a halogen lamp was dispersed using a 0.5-m monochromator. A small fraction of the dispersed light from the monochromator was then split and directed onto a pyroelectric detector to monitor the excitation intensity. The rest of the light was directed onto the sample. A dc voltage was applied to the sample with a series resistor, and the PC signal was detected using a lock-in amplifier. Spectral photoconductivity was measured as a function of temperature under constant illumination intensity. Therefore, to the first approximation, we assume that the photo-generated carrier density is independent of temperature. Therefore, the change in the photoconductivity with temperature is attributed to temperature dependence of the photo-generated carrier mobility.

**Figure 5:**
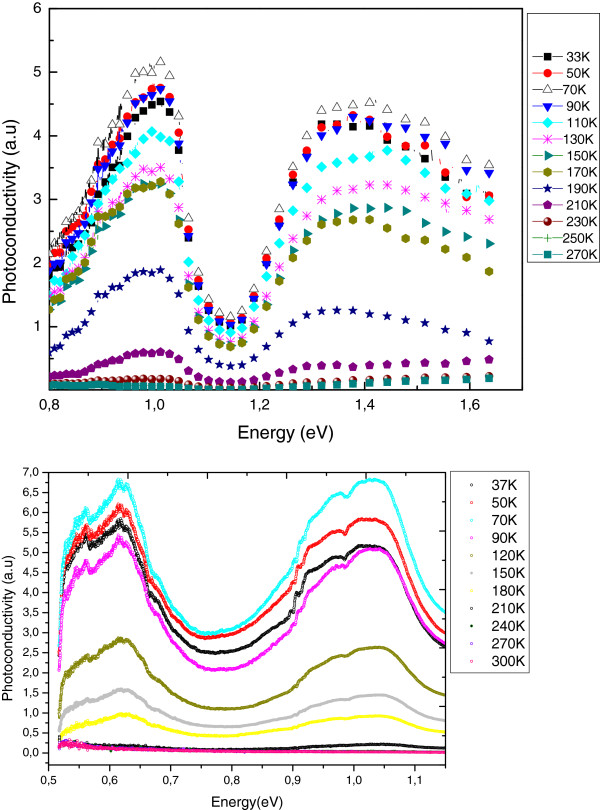
**Mg-doped sample with strong PC.** The measurements were taken using two experimental sets one with a grating for better resolution at high energies (**a**) and the other with a better resolution at low energies (**b**).

There was no detectable PC signal in the undoped material in the range of measurement temperatures (30 >*T* > 300 K). Mg-doped sample gave strong PC where the results are plotted in Figure 5a,b. The measurements were taken using two experimental sets one with a grating for better resolution at high energies (a) and the other with a better resolution at low energies (b). It is clear that the data agree well in the overlapping energy region.

The main features of the PC spectra are the presence of three broad peaks at *E* approximately 0.65 eV (*λ* approximately 1.90 μm), *E* approximately 1.00 eV (*λ* approximately 1.24 μm) and *E* approximately 1.40 eV (*λ* approximately 0.89 μm). The peak at 1.00 eV corresponds to band-to-band transitions with the nominal indium concentration of 80% [31,34]. This is commonly accepted value for the band gap of approximately 1.0 eV for Ga_0.2_In_0.8_ N. The other two peaks may be explained tentatively in terms of a non uniform composition probably associated with indium segregation. The 1.90-μm peak may be due to the Ga-deficient regions with band-to-band transition energy of 0.65 eV corresponding to the InN band gap [34,35]. The higher energy peak at 1.4 eV may be the regions with reduced average indium concentration of 50% [31,34]. The broad nature of the PC peaks does not allow the identification or the existence of the Mg-related transitions.

It is clear from Figure 5 that the peak wavelengths of the PC peaks have no notable temperature dependence. This observation is similar to the behaviour of PL spectra as reported in literature, where there is an extremely weak dependence of the PL peak on temperature [7]. The intensities of PC peaks, however, depend strongly on the temperature of the measurements particularly at temperatures *T* > 90 K where PC drops rapidly with increasing temperature.

**Figure 6:**
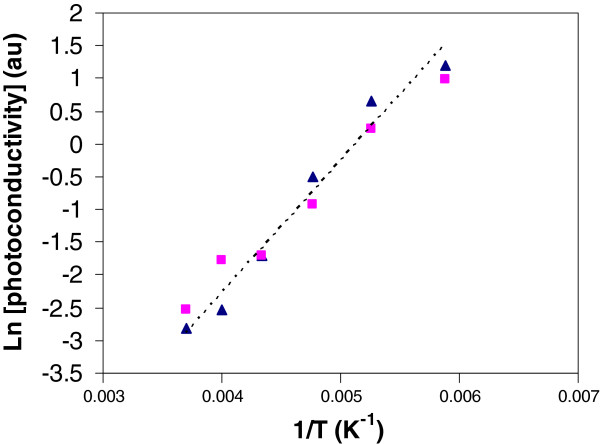
**Logarithm of the peak photoconductivity versus temperature.** Filled triangles, PC peak 1; Filled squares, PC peak 2. The thermally activated behaviour has activation energy of *Φ* = 175 ± 15 meV.

In Figure 6 we plotted the logarithm of the peak photoconductivity against the inverse temperature for the two prominent peaks in Figure 5a, i.e. *E* = 1.0 and *E* = 1.4 eV for temperatures *T* > 150 K. There is a clear thermally activated behaviour in the form

(6) σ∝expΦkT,

with an activation energy of *Φ* = 175 ± 15 meV. In order to attach any significance to this activation energy, we consider the dynamics of photoconductivity. Here we assume that most of the absorption occurs in the bulk layer so that the PC signal is fundamentally associated with the bulk.

Following the theoretical model developed by us for photo-voltage [36], the photoconductivity (*Δσ*) due to the excess electron (*Δn*) and hole (*Δp*) densities is as follows:

(7) $$\Delta \sigma =e\Delta n\mu_{e}+e\Delta p\mu_{n}\text{,}$$

where e, *μ*_*e*_ and *μ*_*h*_ are the electronic charge, electron and hole mobilities respectively.

In the absence of reflection and surface recombination and when the radiation is uniformly absorbed by the sample the rate of generation of electron hole pairs [36,37]

(8) $$ℜ=\frac{q\alpha I}{hv}$$

where *q, α, I* and *hν* are the quantum efficiency, absorption coefficient, intensity of the light absorbed and the photon energy respectively.

In the absence of trapping and in n-type material, the time dependence of the excess hole concentration, i.e. the rate equation is

(9) $$\frac{d\Delta p}{dt}=ℜ-\frac{\Delta p}{\tau_{p}}\text{,}$$

where *τ*_*p*_ is the minority carrier radiative lifetime for holes.

In the steady state

(10) $$\frac{d\Delta p}{dt}=0$$

thus,

(11) $$\begin{array}{c} ℜ=\frac{\Delta p_{0}}{\tau_{p}}={\frac{q\alpha I}{h\nu }}_{p}\\ \Delta p_{0}=\frac{q\alpha I}{h\nu }\tau_{p} \end{array}\text{,}$$

Where *Δp*_0_ is the excess hole density in the steady state.

When the illumination intensity is low or the thermal equilibrium hole density (*p*_0_) is high, i.e.

*Δσ*_0_ < *σ* and *Δp*_0_ < *p*_0_

If there is no trapping, *n*_0_ = *Δp*_0_, the photoconductivity in the steady state becomes

(12) $$\Delta \sigma_{0}=e\Delta n_{0}\mu_{e}+e\Delta p_{0}\mu_{h}=e\Delta p_{0}\left(1+\beta \right)\mu_{h}=eℜ\tau_{p}\left(1+\beta \right)\mu_{h}=e\frac{q\alpha I}{h\nu }\tau_{p}\left(1+\beta \right)\mu_{h}\text{,}$$

where $\beta =\frac{\mu_{e}}{\mu_{h}}$.

Photoconductivity will increase linearly with intensity. However, the experiments were performed at constant illumination intensity. Therefore, at high temperatures (in the optical phonon scattering regime) when the quantum efficiency, absorption coefficient and the recombination lifetime have weak temperature dependences, the temperature dependence of the PC will be determined primarily by the minority carrier mobility.

In the presence of differential trapping (*Δn* ≠ *Δp*), the rate equation for excess holes becomes

(13) $$\frac{d\Delta p}{dt}=ℜ-\frac{\Delta p}{\tau_{p}}-\frac{\Delta p}{\tau_{1}}+\frac{\Delta N_{s}}{\tau_{2}}$$

Here *τ*_*1*_*, τ*_*2*_ and Δ*N*_S_ are the average lifetime before the hole is caught in a trap, average lifetime that a hole stays in the trap (de-trapping time) and the excess hole concentration in the trap.

The time dependence of excess holes in the trap will be

(14) $$\frac{d\Delta N_{s}}{dt}=\frac{\Delta p}{\tau_{1}}-\frac{\Delta N_{s}}{\tau_{2}}$$

In the steady state,

(15) $$\frac{d\Delta N_{s}}{dt}=0\;and\;\Delta N_{s_{0}}=\frac{\Delta p_{0}}{\tau_{1}}\tau_{2}$$

Space charge neutrality requires *Δn*_0_ = *Δp*_0_ + *ΔN*_s0_.

By substituting Δ*N*_s*0*_ from Equation 10, the excess electron density in thermal equilibrium, Δ*n*_*0*_, can be obtained as follows:

(16) $$\Delta n_{0}=\Delta p_{0}\left(1+\frac{\tau_{2}}{\tau_{1}}\right)$$

Thus, the photoconductivity in the steady state:

(17) $$\Delta \sigma_{0}=e\Delta p_{0}\left[\left(1+\frac{\tau_{2}}{\tau_{1}}\right)\mu_{e}+\mu_{h}\right]\text{.}$$

By substituting Δ*p*_0_ from Equation 6, we obtain

(18) $$\Delta \sigma_{0}=eℜ\tau_{p}\left(1+\beta +\beta \frac{\tau_{2}}{\tau_{1}}\right)\mu_{h}\text{.}$$

At high temperatures the temperature dependence of the radiative lifetime and the trapping lifetimes will have weak temperature dependences. Therefore, at high temperatures in the longitudinal-optical (LO) phonon, scattering regime conductivity will be dominated by the product of the two exponential terms, i.e. the de-trapping time [35,38] is

(19) $$\tau_{2}=A\exp\left(\frac{E_{\mathrm{th}}}{kT_{L}}\right)$$

and in the LO scattering limited hole mobility [2,3,25,26]

(20) $$\mu_{h}=\frac{e\tau_{m}}{m^{*}}\exp\left(\frac{\hbar \omega_{\mathrm{LO}}}{kT_{L}}\right)\text{,}$$

where *E*_th_ is the energy difference between the hole trap and the valence band (trap emission energy). m*, *ℏω*_*LO*_, *k*_*B*_, *T*_*L*_*and τ*_*m*_ are respectively, the effective mass, optical phonon energy Boltzmann's constant, lattice temperature and electron-LO phonon scattering (momentum relaxation) time which is given by [2,25,26]

(21) $$\tau m={\left[\frac{e^{2}\omega_{\mathrm{LO}}}{2\pi \hbar }{\left(\frac{m^{*}}{2\hbar \omega_{\mathrm{LO}}}\right)}^{\frac{1}{2}}\left(\frac{1}{\varepsilon_{\infty }}-\frac{1}{\varepsilon_{s}}\right)\right]}^{-1}$$

Here *ε*_*∞*_ = 8.4 *ε*_*∞*_*and* = *ε*_*s*_ = 15.3 *ε*_0_ are the high frequency and static permittivity, respectively.

Therefore, *Δσ*_0_ will have an exponential temperature dependence, i.e.

(22) $$\Delta \sigma_{0}\propto \exp\left(\frac{E_{\text{th}}+\hbar \omega_{\text{LO}}}{kT}\right)$$

At high temperatures a plot of the logarithm of the peak photoconductivity versus inverse temperature should give a straight line with a slope

(23) $$\frac{E_{\mathrm{th}}+\hbar \omega_{\mathrm{LO}}}{k}\text{.}$$

Therefore, the thermal activation energy should correspond to

(24) $$\Phi =E_{\mathrm{th}}+\hbar \omega_{\mathrm{LO}}\text{.}$$

We obtain from Figure 6 that

(25) Φ=175±15meV.

Since the optical phonon energy in indium-rich GaInN is *ℏω*_*LO*_ = 72 *meV*[2,3] the hole traps will have an average depth from the valence band edge of *E*_th_ = 103 ± 15 meV. This value is very close to the Mg binding energy in indium-rich GaInN obtained from the *C-V* and variable magnetic field Hall measurements, in [23,39] and from Figure 3.

**Figure 7:**
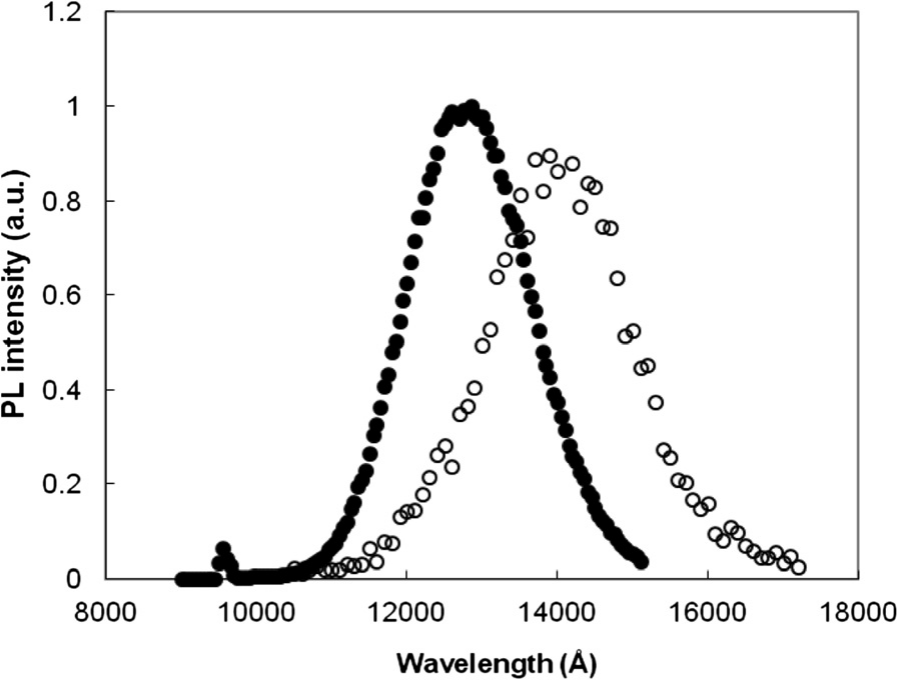
**Normalised PL spectra at*T*= 10 K.** Closed circle, undoped sample (GS 1508). Open circles, Mg-doped sample (GS 1989). The GS 1989 spectrum is amplified by 2 × 10^4^.

In order to confirm the presence of Mg acceptors, we measured the PL spectrum of both doped and undoped material at *T* = 10 K. The results are shown in Figure 7. In the undoped material, the PL due to band-to-band recombination has a peak energy of *E*_B−B_ = 0.98 eV ± 10%. In the Mg-doped sample however, the emission is due to the e-A recombination with the peak energy of *E* = 0.88 eV ± 10%. The difference of Δ*E* approximately 100 meV between the two PL peaks is in the right range for the Mg binding energy and agrees well with the values for the acceptor level obtained from the PC and DC Hall data.

## Conclusions

We have investigated dark (DC) and photo (PC) conductivity and PL in indium-rich GaInN grown by MBE. To our knowledge this is the first spectral measurements of photoconductivity in Mg-doped GaInN. Our results indicate that indium segregation may be responsible for the non uniform composition giving rise to three broad peaks in the photoconductivity spectra. From the temperature dependence of the photoconductivity, an average trap energy of *E*_th_ = 103 ± 15 meV is obtained. Complementary measurements of the transient PL at low temperatures shows e-A transitions with the acceptor binding energy of approximately 100 meV. The temperature dependence of the DC conductivity in Mg-doped GaInN is explained using a multi-channel conduction mechanism, involving p-doped layers in parallel with the n layer, with an acceptor ionisation energy of 108 ± 20 meV. This is also in excellent agreement with the PC and PL results.

## Competing interests

The authors declare that they have no competing interests.

## Authors’ contributions

NB supervised the whole project, developed the model and drafted the manuscript. ET supervised the Hall measurements and carried out PC measurements. AE supervised the PC measurements and checked the manuscript draft. MG carried out the PL measurements. SA carried out the Hall measurements. MCA devised the PC work. DL carried out the transient PL and contributed to the manuscript. HC supervised and analysed the transient PL. XM devised the transient PL and contributed to the modelling. CG analysed the PC results. All authors read and approved the final manuscript.
